# Antiviral efficacy of cerium oxide nanoparticles

**DOI:** 10.1038/s41598-022-23465-6

**Published:** 2022-11-05

**Authors:** Alexandra Nefedova, Kai Rausalu, Eva Zusinaite, Alexander Vanetsev, Merilin Rosenberg, Kairi Koppel, Stevin Lilla, Meeri Visnapuu, Krisjanis Smits, Vambola Kisand, Tanel Tätte, Angela Ivask

**Affiliations:** 1 Institute of Physics, University of Tartu, W. Ostwaldi Street 1, 50411 Tartu, Estonia; 2 Institute of Technology, University of Tartu, Nooruse Street 1, 50411 Tartu, Estonia; 3 Institute of Molecular and Cell Biology, University of Tartu, Riia Street 23, 51010 Tartu, Estonia; 4 Department of Chemistry and Biotechnology, Tallinn University of Technology, Akadeemia Street 15, 12618 Tallinn, Estonia; 5 Institute of Solid State Physics, University of Latvia, Kengaraga Street 8, Riga, 1063 Latvia

**Keywords:** Microbiology, Nanoscience and technology

## Abstract

Nanomaterials are prospective candidates for the elimination of viruses due to their multimodal mechanisms of action. Here, we tested the antiviral potential of a largely unexplored nanoparticle of cerium dioxide (CeO_2_). Two nano-CeO_2_ with opposing surface charge, (+) and (−), were assessed for their capability to decrease the plaque forming units (PFU) of four enveloped and two non-enveloped viruses during 1-h exposure. Statistically significant antiviral activity towards enveloped coronavirus SARS-CoV-2 and influenza virus was registered already at 20 mg Ce/l. For other two enveloped viruses, transmissible gastroenteritis virus and bacteriophage φ6, antiviral activity was evidenced at 200 mg Ce/l. As expected, the sensitivity of non-enveloped viruses towards nano-CeO_2_ was significantly lower. EMCV picornavirus showed no decrease in PFU until the highest tested concentration, 2000 mg Ce/l and MS2 bacteriophage showed slight non-monotonic response to high concentrations of nano-CeO_2_(−). Parallel testing of antiviral activity of Ce^3+^ ions and SiO_2_ nanoparticles allows to conclude that nano-CeO_2_ activity was neither due to released Ce-ions nor nonspecific effects of nanoparticulates. Moreover, we evidenced higher antiviral efficacy of nano-CeO_2_ compared with Ag nanoparticles. This result along with low antibacterial activity and non-existent cytotoxicity of nano-CeO_2_ allow us to propose CeO_2_ nanoparticles for specific antiviral applications.

## Introduction

The search for antiviral agents—materials that enable to inactivate viruses, inhibit their capability to infect their host cells or suppress their ability to replicate^1^, has clearly intensified with the current COVID-19 pandemic^2^. Recently, the potential of nanotechnology in the development of antiviral therapeutics has been acknowledged^3–7^. One of the groups of potential antiviral nanomaterials is metal and metal oxide nanoparticles^8^ that have been suggested to exert their activity via multimodal mechanisms of action^9^, including direct binding to virus surface, inhibiting viral binding to host cells or even interacting with viral genome^10^. Such a broad spectrum of proposed antiviral activities of metal-based nanoparticles may result in a smaller likelihood of developing antiviral resistance, which may occur in case of conventional antiviral drugs^11^.

A wealth of literature has already been published on antiviral nanoparticles. As of January 2022, 1,623 articles were retrieved from ISI Web of Science using keywords “antiviral” AND “nanoparticle*”. Out of those 17% mentioned “COVID” while 30% included “silver”, 5% “copper”, 5% “zinc” and 4% “titanium OR titania”. Interestingly, all those nanoparticles are also among the most utilized nanoparticles in antibacterial applications^12^ indicating that a relatively general mechanism of action, effective against both, bacteria and viruses, may be expected. Nanosilver contributing to 1/3 articles on antiviral nanoparticles, is clearly one of the most studied antiviral nanoparticle types. Potential binding of nanosilver particles onto the outer surface of the viruses and binding of nanoparticles to viral genetic material, leading to further inhibition of virus replication, have been suggested as its modes of action^13^. Efficacy of silver nanoparticles in decreasing the infective viral counts has been demonstrated against a variety of viruses, including HIV^14–17^, herpes simplex virus^18^, influenza virus^19^, noroviruses^20^ adenoviruses as well as SARS -CoV-2^21^ and other viruses^22–25^. It is however worth mentioning that while the antiviral concentrations of silver nanoparticles usually range between tens and hundreds of mg/l^26^, those concentrations may already lead to cytotoxicity^21^ and certainly exhibit antibacterial effect, that usually is evidenced starting from low mg/l range^27^. Indeed, unspecific cytotoxicity and concurrent potential health hazard of some of the proposed nanoparticles may be an issue^28^ and thus, safer nanoparticle alternatives with lower potential health hazards are certainly of interest.

In search for antivirally effective materials with low activity towards human cells or microbiota, we focused on CeO_2_-based nanomaterials. Previously, the antiviral activity of ceria nanoparticles has been suggested in a few papers that concerned influenza virus H1N1 and herpes simplex virus^29,30^, Sabin-like poliovirus^31^ and vesicular stomatitis (VS) virus^30^. Although the mechanism of antiviral activity of CeO_2_ nanoparticles has not been studied thoroughly, some studies suggest that defects in CeO_2_ crystal structure may be responsible for its biological activity^30^. Such defects have been shown to lead to redox reaction Ce(III) ⇔ Ce(IV)of on the surface resulting in filling or formation of oxygen vacancies^32^, and ultimately to the release of reactive oxygen species (ROS) from nanoparticles surface^33^. Interestingly, this formation of ROS by CeO_2_ has been correlated with the acidity of the surrounding environment, thus increasing toxicity in bacteria^34^ as well as cancer cells^31^ at low pH values. In in vitro tests with normal mammalian cells and less acidic pH, no significant cytotoxicity of CeO_2_ nanoparticles has been observed but instead, a protective, survival and growth promoting effect has been demonstrated^35,36^. It has been proposed that these protective effects have been caused by entrapment of free radicals by CeO_2_ and thus, reduction of ROS-induced oxidative stress^37,38^. This combination of potential antiviral activity and low general toxicity suggests that CeO_2_ materials may be indeed potent antivirals with low side effects.

The aim of this work was to reveal the antiviral activity of ultrasmall CeO_2_ nanoparticles towards a selection of enveloped and non-enveloped mammalian viruses and selected bacteriophages. In parallel to CeO_2_ nanoparticles we tested the antiviral activity of nanoparticles of silver that are generally considered to exhibit a significant antiviral activity, and silica (SiO_2_) presumably biologically inert^39^. To reveal the cause of antiviral activity of CeO_2_ nanoparticles, we studied the potential surface protein inactivation by those nanoparticles using SARS-CoV-2 as an example. Finally, the antiviral potency of CeO_2_ and other selected nanoparticles was compared with their bactericidal activity towards Gram-negative *Escherichia coli* and Gram-positive *Staphylococcus aureus* and cytotoxic effects.

## Results and discussion

### Characterization of nanoparticles

Although the emphasis of this work was on antiviral effects of CeO_2_ nanomaterials, nanoparticles of Ag and SiO_2_ were added to the testing as controls for a generally approved antiviral agent and an inert nanoparticle (Table 1). Acknowledging the importance of nanoparticles surface in their biological effects, nano-CeO_2_ were synthesized with two different states of surface, those with nearly “bare” surface carrying large positive charge (nano-CeO_2_(+)) and nanoparticles stabilized by citric ions and, thereby presenting negatively charged surface (nano-CeO_2_(−)). Nano-CeO_2_(+) were synthesized at maximal concentration of 6500 mg Ce/l (ca. 45 mM) and nano-CeO_2_(−) at 8200 mg Ce/l (ca. 60 mM). Hereafter, in described experiments, mg/l concentration for investigated compounds always refers to milligrams per liter of the element, i.e. Ce, Ag or Si.

**Table 1:** Key characteristics of nanoparticles used.

Nanoparticle	TEM size, nm	Hydrodynamic size^a^, nm	ζ potential, mV
nano-CeO_2_(+)	3.3 ± 0.4	7.0 ± 3.0	+ 41 ± 2
nano-CeO_2_(−)	3.2 ± 0.4	4.5 ± 2.0	− 53 ± 4
nano-Ag	15.3 ± 3.0	18.5 ± 3.0	− 52 ± 5
nano-SiO_2_	16.6 ± 1.9	45 ± 5	− 34 ± 3

^a^Number based hydrodynamic size according to DLS (dynamic light scattering) measurements.

**Figure 1:**
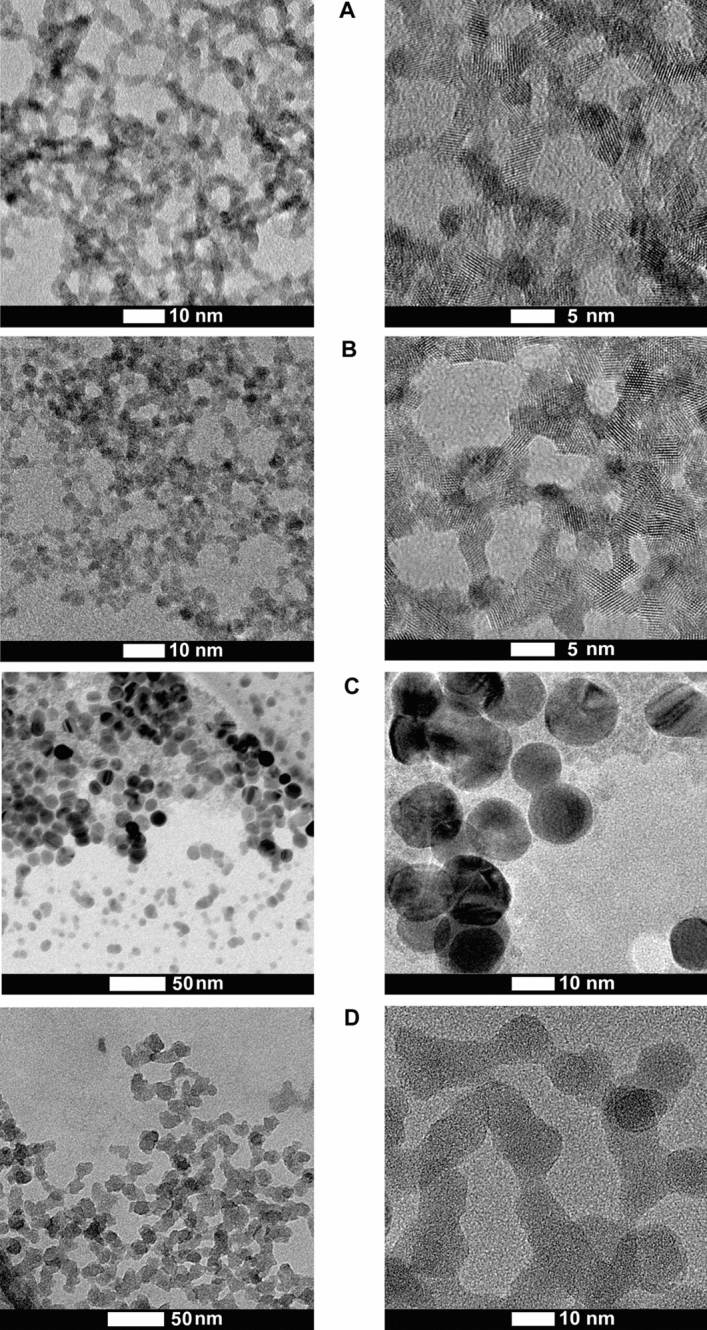
HRTEM images of synthesized nanoparticles. (**A**) Nano-CeO_2_(+), (**B**) nano-CeO_2_(−), (**C**) nano-Ag, (**D**) nano-SiO_2_.

As synthesized, nano-CeO_2_(+) and nano-CeO_2_(−) formed stable aqueous colloids due to their large (+ 41 mV or − 53 mV, respectively) ζ-potential (Table 1). According to high resolution transmission electron microscopy (HRTEM) (representative TEM images of CeO_2_ nanoparticles are shown on Fig. 1), the average particle size of both, nano-CeO_2_(+) and nano-CeO_2_(−) was close to 3 nm (Fig. S1). Dynamic light scattering-measured hydrodynamic size of nano-CeO_2_(−) determined at highest tested antiviral concentration was relatively close to the particles primary size (Table 1) and only nano-CeO_2_(+) showed some aggregation. Interestingly, with decrease in nano-CeO_2_ concentration apparent increase in particle size and thus, the likelihood for aggregation was observed with decreasing particle concentration for both, nano-CeO_2_(+) and nano-CeO_2_(−), whereas the latter was more sensitive to dilution-mediated instability (Fig. S2). We suggest that desorption of stabilizing citric ions from the surface of nanoparticles upon dilution of colloid lead to the thinning of nanoparticles double electric layer and deteriorated colloidal stability.

**Figure 2:**
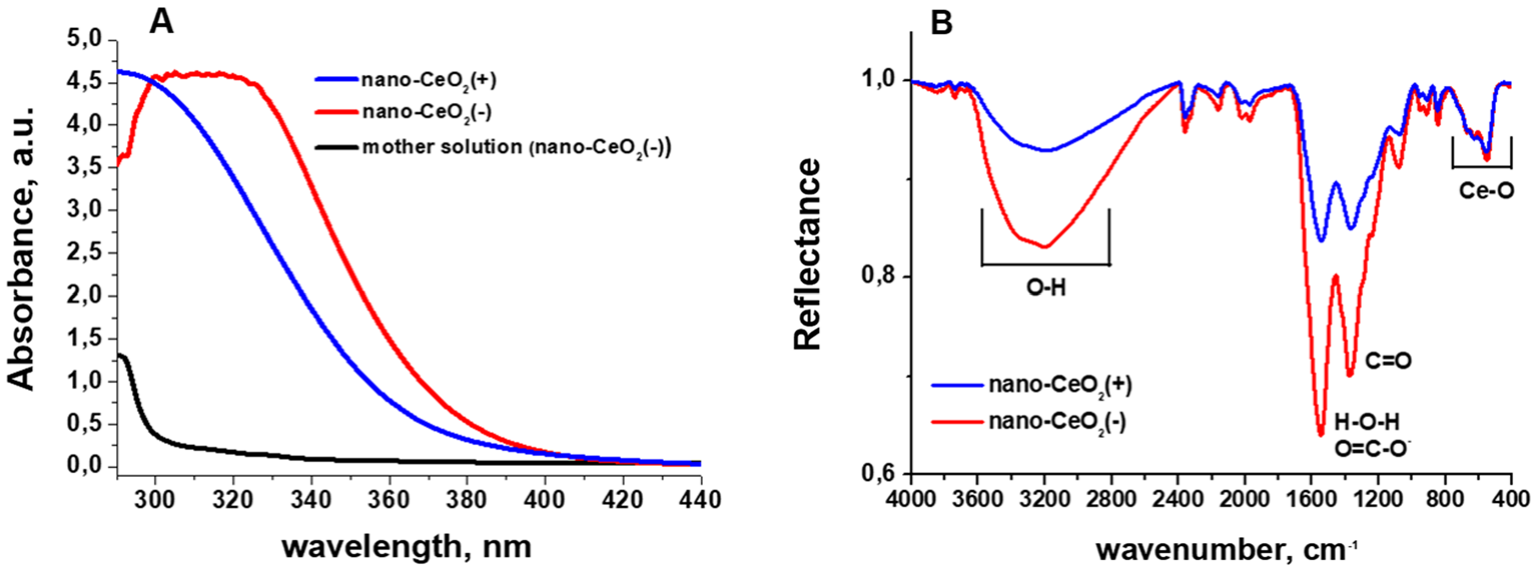
Characteristics (**A**) UV–Vis spectra and (**B**) infrared spectra of nano-CeO_2_(+) and nano-CeO_2_(−). On (**A**) also the UV–Vis spectrum of nano-CeO_2_(−) mother solution is shown.

UV–Vis spectra of CeO_2_ nanoparticles (Fig. 2A) showed that compared to nano-CeO_2_(+) the fundamental absorption edge of nano-CeO_2_(−) was approximately 10 nm red shifted. Also, visibly, the colloid of nano-CeO_2_(−) showed a deeper yellow color compared to nano-CeO_2_(+). This difference in shift of absorption edge may be due to the different Ce(III)/Ce(IV) ratio on the surface of nanoparticles, or originate from oxidized and polymerized citrate ions on the surface of nano-CeO_2_(−) particles, as a remnant of the synthesis process. The seeming “drop” of the absorption of nano-CeO_2_(−) colloid below 300 nm is a compensation error due to a symmetric rise of absorption of the mother solution (the liquid remaining after centrifugation of nanoparticles) of this colloid caused, most likely, by a high concentration of citrate ions and products of their oxidation and/or complexing with cerium ions. Infrared (IR) spectrum of the synthesized nano-CeO_2_ (Fig. 2B) is in agreement with the literature data^40^ and exhibits peaks in around 3600 to 3200 cm^−1^, which correspond to –OH stretching of absorbed water and surface OH-groups. Peaks around 1600 cm^−1^ and 1400 cm^−1^ are H–O–H deformational vibrations and C–O stretching vibrations from surface water molecules and carbonate ions, respectively. The peak from 700 to 500 cm^−1^ is due to Ce–O stretching vibrations. The differences between nano-CeO_2_(+) and nano-CeO_2_(−) spectra are mainly intensities of peaks around 1600 cm^−1^ and 1400 cm^−1^, which in case of nano-CeO_2_(−) correspond also to COO– group vibrations of absorbed citrate ions^41^. Also we observed a small blue-shift of the band from 1539 cm^−1^ nano-CeO_2_(+) to 1546 cm^−1^ for nano-CeO_2_(−), which is probably due to summing of H–O–H deformational vibrations with anti-symmetric COO– group stretching band with maximum should be around 1585 cm^−1^ according to literature data^41^. Together with DLS data, UV–Vis and IR-spectroscopy clearly show, that despite very close mean particle size, shape, and size distribution parameters, nano-CeO_2_(+) and nano-CeO_2_(−) particles are chemically and electrostatically rather different and, therefore, we can expect from them different effect on biological objects. The experimentally established (by means of ICP-MS) concentrations of Ce^3+^/Ce^4+^ ions in supernatants from 1.4 × 10^–2^ M (2000 mg Ce/l) CeO_2_ colloids were rather low: 8 × 10^–5^ M (12 mg Ce/l) for CeO_2_(+) and 1.7 × 10^–4^ M (24 mg Ce/l) for CeO_2_(−). These values are yet significantly higher than literature data^42^, which should be in the range of 10^–7^–10^–8^ M, in water at neutral pH range, and probably are the result of incomplete separation of the nanoparticles. However, even measured concentration should have minimal effect on the viruses.

The synthesis of nano-Ag was carried out through seed-mediated citrate route and the maximum concentration reached was 480 mg/l (corresponds to 4.4 mM). Nano-SiO_2_ was synthesized using Stöber technique and the maximum concentration reached for amorphous silica nanoparticles was 3100 mg/l (corresponds to 110 mM). Both, nano-Ag and nano-SiO_2_ particles were spherical with average primary size around 15 nm (Table 1). According to hydrodynamic size and DLS analysis, nano-Ag remained in non-aggregated state while nano-SiO_2_ exhibited a certain level of aggregation and formation of 2–10 nanoparticle aggregates. The surface ζ-potential of nano-Ag and nano-SiO_2_ particles was negative (− 52 ± 5 and − 34 ± 3 mV, respectively) being comparable to nano-CeO_2_(−), thus allowing the comparison of their biological effects. Unfortunately, we were unable to synthesize neither silica nor silver nanoparticles with primary size comparable to CeO_2_ nanoparticles without the addition of strong surfactants that would have their own pronounced antimicrobial properties.

### Antiviral activity of CeO_2_ nanoparticles

For the demonstration of antiviral potency of nano-CeO_2_, nano-SiO_2_ and nano-Ag, we analyzed the decrease of infective counts (expressed as plaque forming units, PFU) of four mammalian viruses and two bacteriophages after 1-h contact with nanoparticles. Nano-CeO_2_ with both positive and negative surface ζ potentials were used to understand the effect of CeO_2_ surface in their antiviral activity. To rule out the possibility that the antiviral activity is only due to the nanometer scale particle size, parallel antiviral activity testing was performed with supposedly biologically inert, low toxicity SiO_2_ nanoparticles. Conversely, nano-Ag particles that are widely considered to exhibit antiviral effect, were tested as positive controls in antiviral tests (see Table 2). Despite the very low potential CeO_2_ solubility, we also tested the antiviral effect of soluble Ce(III) compound Ce(NO_3_)_3_ as a source of Ce^3+^ ions_._ Although both, Ce^3+^ and Ce^4+^ ions could form at low concentration during the dissolution of CeO_2_, Ce^3+^ ions were used due to their higher likelihood of presence^42^.

**Table 2:** Characteristics of viruses used in antiviral assays.

Virus	Characteristics	Nanoparticle or compound*	Purpose	Concentration range tested
Influenza A/WSN/1933	Enveloped virus, family *Orthomyxoviridae*, seasonally important to cause flu in Northern hemisphere	Nano-CeO_2_(+)	Proposed antiviral compound	0.002–2000 μg Ce/ml**
SARS-CoV-2	Enveloped virus, family *Coronaviridae*, causes COVID-19	Nano-CeO_2_(−)	Proposed antiviral compound	0.002–2000 μg Ce/ml**
TGEV	Enveloped virus, family *Coronaviridae*, transmissible gastroenteritis virus of pigs	Ce(NO_3_)_3_	Control for Ce(III) ion effects	0.002–2000 μg Ce/ml**
EMCV	Non-enveloped virus, family *Picornaviridae* causative agent for neurological disorders	Nano-Ag	Nanoparticle with previously demonstrated antiviral activity	0.0015–1500 μg Ag/ml**
Ф6	Enveloped bacteriophage of *Pseudomonas* sp.	Nano-SiO_2_	Proposed inert nanoparticle	0.0004–400 μg Si/ml**
MS2	Non-enveloped bacteriophage of *Escherichia coli*			

Right side of the table shows characteristics and concentrations of nanoparticles and chemical compounds used in antiviral assays with every virus listed in the left side of the table.

*All the compounds were tested with all the viruses.

**All the tested nanoparticles and chemicals were in the range of 0.014–14,000 μM.

Our selection of viruses included mammalian and bacterial, both enveloped and non-enveloped viruses (Table 2). In general, enveloped viruses surrounded by a lipid membrane have been considered more sensitive to inactivation by various environmental conditions than non-enveloped viruses possessing only a proteinaceous capsid^43^. Pathogenic viruses can belong to either of these groups, therefore, most of the antiviral testing standards foresee the inclusion of both types of viruses^44,45^. The enveloped mammalian viruses used in this study included influenza virus^46^, SARS-CoV-2 and TGEV^47^, and picornavirus EMCV was used as a model of the non-enveloped mammalian viruses^48^. Enveloped Ф6^49^ and non-enveloped MS2^50^ were used as respective bacterial virus (bacteriophage) examples. Both of those phages have been suggested as models for antiviral testing^51–54^. In case of all the viruses, exposure to nanoparticles was carried out in sterile water or highly diluted growth medium over 1 h. The numbers of infectious viral particles with and without treatments were expressed as plaque forming units (PFU/ml).

Prior to antiviral activity testing, the effect of all the tested compounds was studied on viral host cells. Most of the tested compounds and concentrations were non-cytotoxic to the host cells of mammalian viruses (Fig. S3). Only the highest tested concentrations of nano-Ag, Ce(NO_3_)_3_ or SiO_2_ decreased the viability of some of the host cell lines, which however did not interfere with antiviral assays, where the concentrations of those compounds did not reach the toxic levels. Except for nano-Ag none of the compounds affected the growth of host bacteria of bacteriophages. As nanosilver is well known for its antibacterial effects, its bacterial toxicity that was observed from 14 μM (1.5 mg/l) was not a surprise. To be able to study the effects of nano-Ag towards bacteriophages, its bacterial toxicity was neutralized by the addition of threefold molar excess of l-cysteine to the infection reaction immediately prior to the plaque assay step.

**Figure 3:**
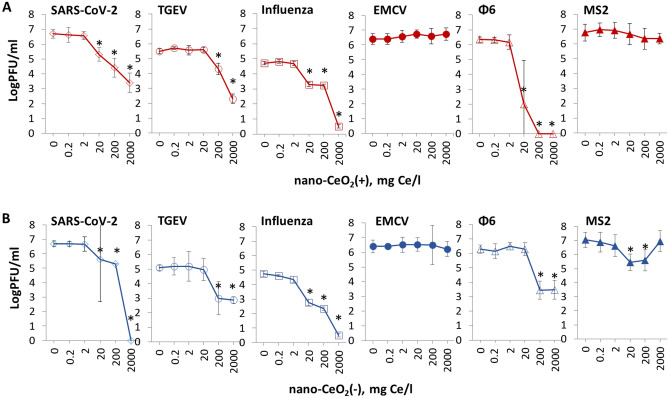
The effect of nano-CeO_2_(+) (**A**) and nano-CeO_2_(−) (**B**) on viral plaque forming activity (log PFU/ml). Enveloped mammalian viruses and bacteriophages are shown with open symbols; closed symbols represent non-enveloped mammalian viruses and bacteriophages. The differences in initial viral titers are caused by different viral yields in laboratory conditions. *p < 0.05.

The results of antiviral assay showed that despite being non-toxic to mammalian cells and bacteria, nano-CeO_2_ affected the infectivity of most of the mammalian viruses and bacteriophages (Fig. 3). Significant decrease (p ≤ 0.05) of viral infectivity due to 1-h nano-CeO_2_(+) exposure was observed starting from 20 mg/l in case of SARS-CoV-2, influenza virus and ф6, and starting from 200 mg/l in case of TGEV (Table 3). None of the non-enveloped viruses showed significant decrease in infectivity for nano-CeO_2_(+) up to 2000 mg/l. Nano-CeO_2_(−) affected the infectivity of SARS-CoV-2 and influenza virus starting from 20 mg/l and the infectivity of TGEV and ф6 starting from 200 mg/l (Table 3). Following the trend of nano-CeO_2_(+), non-enveloped viruses showed the lowest sensitivity towards nano-CeO_2_(−). EMCV infectivity was not affected by nano-CeO_2_(−) up to 2000 mg/l, and the response of non-enveloped bacteriophage MS2 to nano-CeO_2_(−) was non-monotonic so that 20 and 200 mg/l of the compound decreased the infectivity, but no effect was observed at 2000 mg/l. Such a non-monotonic effect on antiviral activity could not be explained by increasing aggregation level of CeO_2_ at higher concentration as according to hydrodynamic size measurement, as nano-CeO_2_ were non-aggregated at higher concentrations and particle aggregation and instability increased with dilution level of the particles (Fig. S2). Non-monotonic response has been shown also for other types of nanomaterials. For example, TiO_2_ exhibits non-monotonic UV light-induced toxicity to freshwater organisms^55^. In case of CeO_2_ nanoparticles, earlier reports can be found on non-monotonic toxicity towards soybean plants^56^, however, its exact causes are still to be clarified.

**Table 3:**
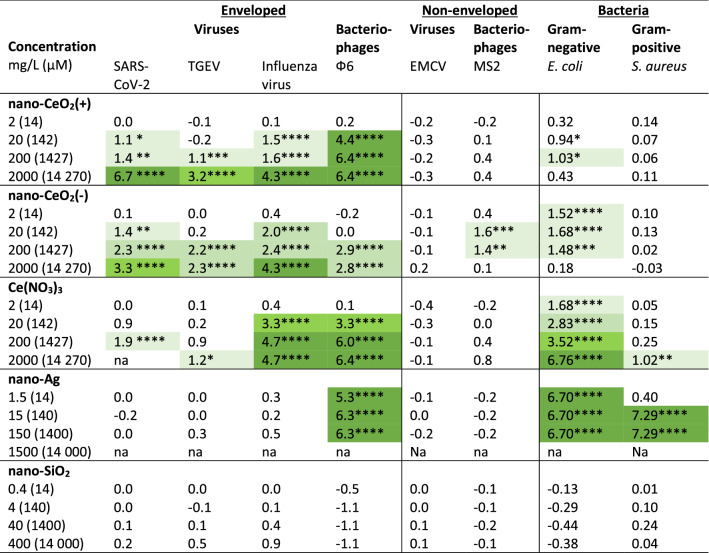
Antiviral or bactericidal activity at different concentrations of nanoparticles and Ce(NO_3_)_3_ (numerical data from Figs. 4, 6).

Antiviral activity is expressed as log reduction in infectious viral titer compared to unexposed control after 1 h. Bactericidal activity is expressed as log reduction in viable counts compared to unexposed control after 1 h. Concentrations causing at least 1 log (90%), 2 logs (99%), 3 logs (99.9%) and 4 logs (99.99%) decrease are indicated with light to dark green color gradient. A minimum of 2 log reduction (99%) in viable bacterial count or infectious viral titer is generally considered as lowest biologically meaningful activity in antimicrobial applications while at least 4 log reduction within up to 1 h is required from biocidal products in suspension tests in the framework of European legislation^64^.

*p < 0.05, **p < 0.01, ***p < 0.001 and ****p < 0.0001.

Although we proposed that due to the chemical and electrostatic differences between the surface of nano-CeO_2_(+) and nano-CeO_2_(−) those nanoparticles may exhibit different biological effects, our results showed no notable differences between the antiviral properties of those two types of particles (Table 3).

Comparison of our results on antiviral properties of nano-CeO_2_ with earlier reports is rather difficult due to the variable nature of the nanoparticles, as well as methods used for antiviral activity assessment. However, certain comparisons may be made. For example, in a 2010 study, 2 log reduction of enveloped *Enterobacter aerogenes* infecting bacteriophage UZ1 PFUs was observed after its 2-h exposure to 50 mg/l CeO_2_ whereas 500 mg/l of CeO_2_ was sufficient to decrease infectivity by 4 logs^57^. Mohamed et al.^31^ showed that 14 nm sized nano-CeO_2_ at concentrations less than 50 mg/l removed all the infectious viral particles (PFU) of type 1 Sabin-like poliovirus and the authors even suggested CeO_2_ nanoparticles as an alternative to treatment of polio infection. Yet there are also reports showing no effect by CeO_2_ nanoparticles on viruses. According to Neal et al.^58^ 200 mg/l of CeO_2_ nanoparticles did not have any significant effect on infectivity of human coronavirus OC43 or rhinovirus RV14 after 6 h incubation. However, after doping of CeO_2_ with Ag, antiviral activity of CeO_2_ nanoparticles was achieved, according to authors, due to the presence of increased proportion of Ce(III) ions on the nanoparticles surface, as well as the presence of silver nanoparticles, their size, morphology and the density of their population on CeO_2_ surface. Overall, these few articles published so far on antiviral efficacy of CeO_2_ have demonstrated relatively variable results, which may be attributed to differences in physico-chemical properties of nanoparticles, to different viruses used or antiviral assays applied. However, based on the few earlier studies demonstrating antiviral effects of nano-CeO_2_^31,57^ and the results of our study, we may suggest that nanoceria has a significant potential in antiviral treatments and that this potential should be studied and developed further.

**Figure 4:**
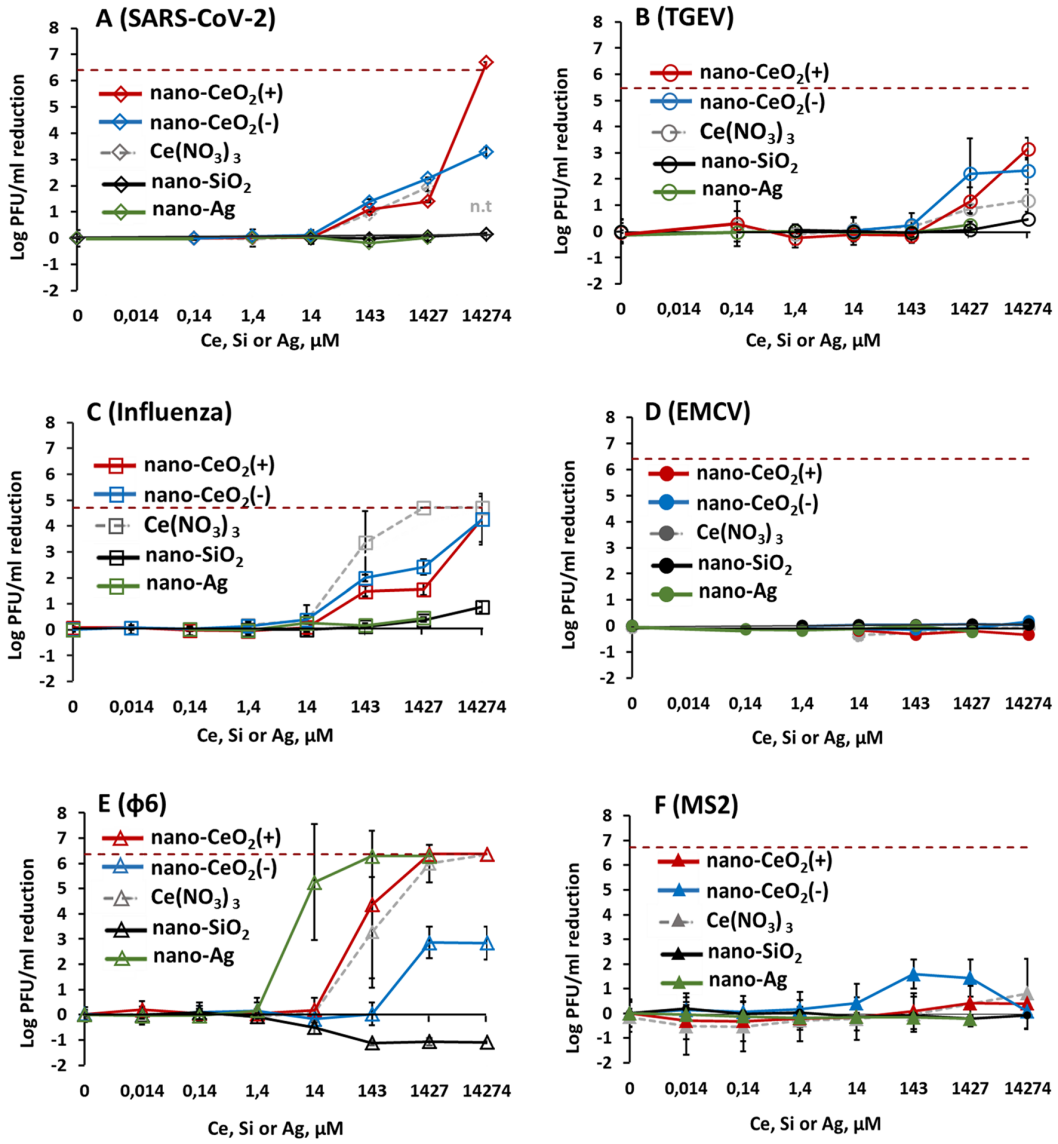
Antiviral activity of all tested nanoparticles and chemical compounds expressed in µM concentrations to enable comparison. Please note that antiviral activity is expressed as reduction of infectious viral titer, logPFU per ml compared to unexposed control after 1 h. The data of nano-CeO_2_ are transferred from Fig. 3. (**A**) coronavirus SARS-CoV-2; (B) transmissible gastroenteritis virus TGEV; (**C**) influenza virus A/WSN/1933; (**D**) picornavirus EMCV; (**E**) bacteriophage ф6; (**F**) bacteriophage MS2. Closed symbols in (**D,F**) represent non-enveloped viruses. The results of three independent experiments with standard deviation are shown. Note the logarithmic scale of y-axis. Horizontal dark red dashed line shows the limit of quantification of PFU reduction**.**

Comparison of the antiviral efficacy of nano-CeO_2_ in comparison with nano-SiO_2_ that was used as a negative control, and nano-Ag used as a positive control due to its likely antiviral properties are shown in Fig. 4. Expectedly, the non-enveloped viruses that did not show any sensitivity towards nano-CeO_2_ were also not influenced by nano-SiO_2_ or nano-Ag (Fig. 4D,F). The inexistent antiviral effect of SiO_2_ nanoparticles was expected as silica is considered biologically compatible or generally regarded as safe^59,60^ and of low toxicity. We found no published data that would have indicated an antiviral activity of SiO_2_ nanoparticles. Yet silica nanoparticles have been shown to impede antiviral response to norovirus infection, reducing the viability of macrophages^61^.

While the low antiviral activity of SiO_2_ was anticipated, our results indicating no significant antiviral activity of nano-Ag up to 150 mg/l (1.4 mM), except for bacteriophage ф6 (Table 2) were surprising. Earlier studies have suggested that nanosilver may affect viruses in a variety of ways; through interactions with viral surface and subsequently interfering the viral attachment to its targets, inhibition of viral penetration into host cells or even by interacting with viral genome^26^, and that silver nanoparticles are affecting viruses already starting from tens of mg/l. However, interestingly, a closer look to Ag nanoparticles antiviral results revealed that in most of the studies only a relatively small, less than 1 log (< 90%) decrease in infectivity has been observed. Yadavalli et al.^8^ have shown inhibition of 50% HIV by Ag nanoparticles between 25 and 5000 mg/l, Rogers et al.^24^ have demonstrated 60–80% decrease in monkeypox virus plaque formation activity in the presence of 20–2000 mg/l of Ag nanoparticles. Up to 90% inhibition of human parainfluenza virus in the presence of 0.1–9 mg/l Ag nanoparticles has been demonstrated by Gaikwad et al.^62^. Slightly higher, up to 2 log decrease of influenza virus titer was observed in response to 50–70 mg/l Ag nanoparticles treatment^63^ and Castro-Mayorga et al.^20^ demonstrated 4 log decrease of median tissue culture infectious dose of feline calicivirus after exposure to 10 mg of nano-Ag/l. Considering that the decrease of viral titers ≥ 2 log can be regarded as the lowest biologically meaningful activity in antimicrobial applications and at least 4 log reduction within up to 1 h is required for biocidal products in suspension tests in the framework of European legislation^64^, there is only little objective proof that Ag nanoparticles would be effective antivirals from the point of view of these requirements. Therefore, our conclusion on low antiviral activity of nano-Ag in terms of log PFU decrease during 1-h exposure (Table 2) is at broad level in line with the previous reports. However, in general view this conclusion is rather surprising as it does not prove the antiviral efficacy of nanosilver contrary to the common belief.

### The mechanism of antiviral activity of nano-CeO_2_

There is no clear mechanism of action proposed as the basis of antiviral activity of CeO_2_ nanoparticles. The elemental analysis of the mother solution compared with literature data allowed us to exclude the possibility of the action of CeO_2_ nanoparticles via released Ce ions^65^. The equilibrium concentration of released ions was found to be extremely low (10^–7^–10^–8^ M)^42,66^ and our experiments with Ce(NO_3_)_3_ showed that the antiviral activity Ce^3+^ ions can be only evidenced at concentrations several orders higher than achievable due to dissolution of CeO_2_ nanoparticles (Fig. 4).

As a different mechanism of inactivating viruses, binding of CeO_2_ nanoparticles on viral genetic material has been reported. Link et al.^67^ showed high binding capacity of nanoparticles of CeO_2_ for nucleic acids in adeno-associated virus, adenovirus, human immunodeficiency virus, and murine leukemia virus^67^. Binding of nanoparticles onto the genetic material of viruses could however take place only when the latter is non-protected by the capsid, e.g., during replication. Another mode of binding has been proposed by Neal et al.^58^, who suggested that Ag-doped CeO_2_ nanoparticles may physically interact with the membrane of enveloped viruses, thus leading to the disruption of their lipid bilayer. In case of non-enveloped viruses, an interaction with virion proteins was suggested.

**Figure 5:**
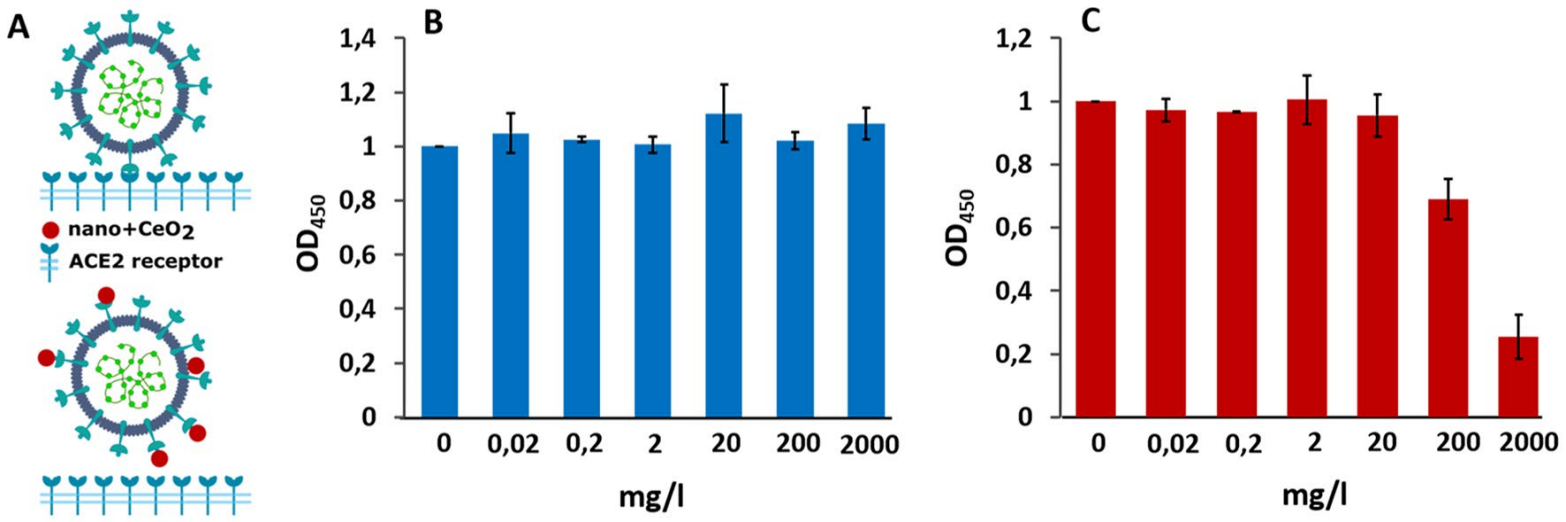
Schematics of the experiment (**A**), where the upper part shows SARS-CoV-2 binding to ACE2 receptor without nanoparticles and lower part demonstrates the theoretical inhibition of SARS-CoV2 binding to ACE2 receptor by of CeO_2_ nanoparticles. (**B**) Shows the effect of nano-CeO_2_(+) and (**C**) the effect of nano-CeO_2_(−) particles on binding of SARS-CoV-2 onto ACE2 receptor in an ELISA assay, measured as optical density (OD450).

As our antiviral assay involved exposure of whole virions to nanoparticles prior to cell infection, we analyzed whether nano-CeO_2_ could affect the binding of a virus to its natural ligand. ELISA assay was performed to measure the in vitro binding activity of nano-CeO_2_ exposed SARS-CoV-2 to its cellular target, the human recombinant ACE2 receptor (Fig. 5). Interestingly, exposure of SARS-CoV-2 to nano-CeO_2_(−) and nano-CeO_2_(+) affected the virus somewhat differently. While exposure of SARS-CoV-2 to ≥ 200 mg/l of nano-CeO_2_(−) inhibited the binding of the virus to ACE2, exposure of SARS-CoV-2 to nano-CeO_2_(+) had no observable effect. However, as the antiviral profiles of both CeO_2_ nanoparticles were relatively similar, these results do not allow us to claim that surface binding and blocking of ligand binding are the leading mechanism of antiviral action of nano-CeO_2_ evidenced by us in antiviral assays. Therefore, the mode of action driving the antiviral effect of CeO_2_ nanoparticles is to be elucidated in future studies.

### Bactericidal effect of CeO_2_ nanoparticles

**Figure 6:**
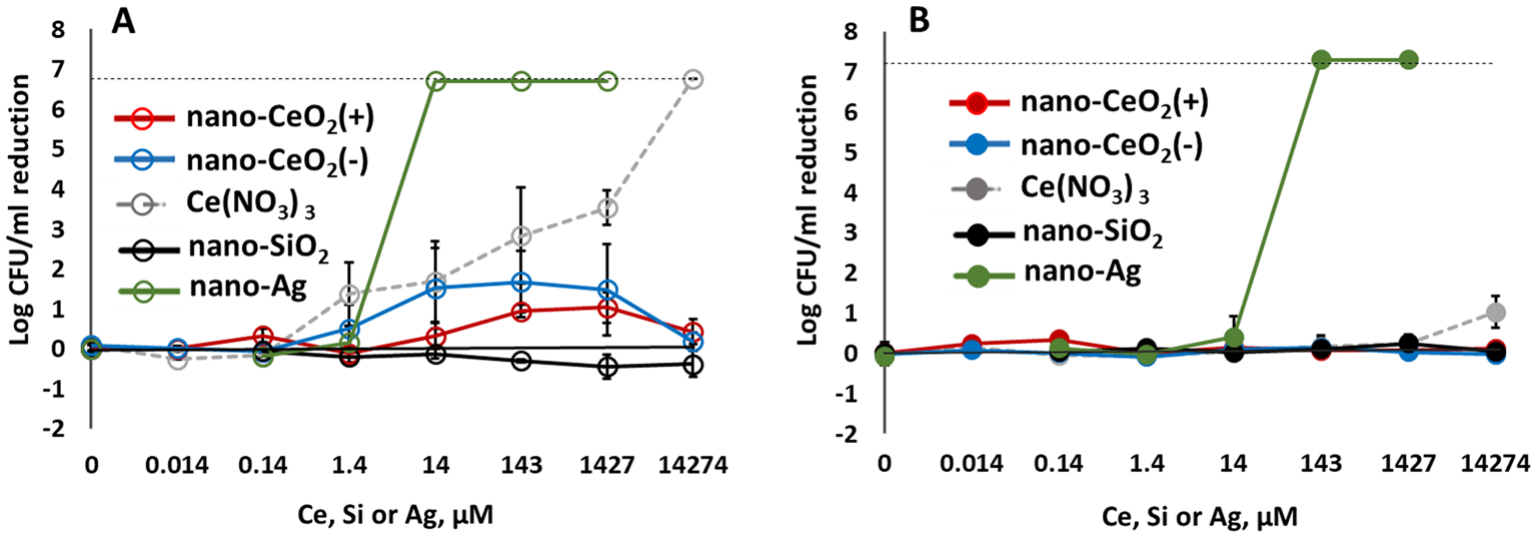
Bactericidal activity after 1 h exposure to nano-CeO_2_, Ce(NO_3_)_3_, nanoparticles of SiO_2_ and Ag against Gram-negative *Escherichia coli* (**A**) and Gram-positive *Staphylococcus aureus* (**B**). Bactericidal activity is expressed as log reduction in viable counts compared to unexposed control after 1 h. Results of three independent experiments with standard deviation are expressed as reduction of log CFU/ml. The dotted line indicates a limit of quantification.

In parallel to antiviral properties, antibacterial activity towards the Gram-negative *Escherichia coli* and Gram-positive *Staphylococcus aureus* was analyzed. According to our results, neither nano-CeO_2_(+) nor nano-CeO_2_(−) up to 2000 mg/l decreased the viable counts (expressed as colony-forming units, CFU) of *E. coli* or *S. aureus* by more than 2 logs (99%) (Fig. 6, Table 3), indicating that the antiviral effect of CeO_2_ nanoparticles was more pronounced than their bactericidal efficacy. Comparison of smaller changes than 2 log decrease in CFU levels in *E. coli* and *S. aureus* showed that Gram-negative *E. coli* was affected by nano- CeO_2_ to a higher extent than Gram-positive *S. aureus* (Table 3). While nano-CeO_2_(−) concentrations of 2, 20 and 200 mg/l decreased the number of *E. coli* CFUs by 1.48–1.68 logs, no decrease in viable counts of *S. aureus* in response to nano-CeO_2_ was observed. Interestingly, the response of *E. coli* was non-monotonous as also in case of non-enveloped bacteriophage MS2. The higher sensitivity of Gram-negative bacteria compared with Gram-positive bacteria against nano-CeO_2_ particles has been shown also in earlier studies and has been related to redox activity of nanoceria and the ability of a thicker peptidoglycan layer to alleviate this effect^68^. In the literature, variable results can be found on antibacterial effects of CeO_2_ nanoparticles, both due to the variety of nanoparticles used as well as due to the different antibacterial assays applied. The minimal inhibitory concentrations (MIC) of nano-CeO_2_ towards a series of Gram-positive and -negative bacteria has been shown to vary between 20 and 140 mg/l^69,70^. Another study where nanoparticles of CeO_2_ were prepared using surfactant Tween-80, demonstrated MIC of 150 mg/l whereas without the surfactant the MIC value was 3000 mg/l^71^. In another study, complete inactivation of *E. coli* was achieved in the presence of 1000 mg/l of CeO_2_ nanoparticles^72^. Thus, our result showing no significant antibacterial activity of CeO_2_ towards Gram-positive *S. aureus* (Table 2) and relatively modest antibacterial effect towards Gram-negative *E. coli* up to 2000 mg/l, coincided with some of the results published earlier but differed from others, respectively.

Differently from nano-CeO_2_ that showed significant or relatively modest antibacterial effect, nano-Ag particles demonstrated significant antibacterial effect already at 1.5 mg/l. Expectedly Gram-negative bacteria were more sensitive towards nano-Ag and at 1.5 mg/l, already more than 4 logs decrease in *E. coli* viable counts was observed. Viability of Gram-positive *S. aureus* decreased by > 4 log from 15 mg nano-Ag/l (Fig. 6, Table 3). These results are in agreement with earlier studies that have shown the efficacy of Ag nanoparticles starting from low mg/l range^27^. Indeed, silver nanoparticles that are shown to act via silver ion release and ROS formation^73^ and the following interaction between Ag ions and thiol groups of proteins as well as permeabilization of bacterial membrane, have been generally regarded as the nanoparticles with highest antibacterial activity. In January 2022, more than 18,000 articles were registered in ISI WoS on “nano* AND silver* AND antibacter*”.

Nanoparticles of SiO_2_, generally regarded as harmless, did not show any bacterial toxicity in our experiments. Inexistent antibacterial activity of nano-SiO_2_ alone has been shown also in other studies, however, most of the papers on SiO_2_ used silica nanoparticles as carriers of more biologically active metal ions or other nanoparticles and thus, are not an adequate comparison for our purpose.

## Conclusion

In this study, we analyzed the decrease of viral infectivity due to exposure to CeO_2_ nanoparticles. According to a few earlier studies, CeO_2_ materials may exhibit significant antiviral activity while being relatively harmless towards human cells and even protecting cells from environmental stress. This suggests that CeO_2_ nanoparticles could be considered as promising antivirals. Our analysis of the activity of two types of CeO_2_ nanoparticles towards four enveloped viruses demonstrated more than 2 logs (99%) decrease in viral infectivity after exposure to 20–200 mg Ce/l of nano-CeO_2_ and more than 4 logs (99.99%) decrease in infectivity after exposure to 200–2000 mg/l. The two non-enveloped viruses were expectedly less sensitive towards nano-CeO_2_; one of them, the EMCV picornavirus showed no decrease in infectivity until the highest tested concentration and the other, MS2 bacteriophage, demonstrated a slight non-monotonous decrease in infectivity after exposure to one of the tested CeO_2_ particles. This allows us to hypothesize, that CeO_2_ nanoparticles may interact not solely with proteins, but also with other components of the viral envelope, e.g. phospholipids, that are absent in the case of non-enveloped viruses.

Interestingly, even if the two CeO_2_ nanoparticles exhibited different surface properties, one carrying a positive surface charge and the other negatively charged citrate group on its surface, their antiviral activity was relatively similar. This allows us to suggest that the intrinsic properties of the nanoparticles rather than surface charge or surface functional groups were responsible for the observed antiviral effects. Moreover, our results indicating relatively low antiviral activity of Ce-ions and inexistent toxicity of an inert nanoparticle of SiO_2_ allow us to conclude that the antiviral activity of nano-CeO_2_ was not due to the release of ions or specific effects by nanosized materials. Compared with viruses, bacteria showed significantly lower sensitivity towards CeO_2_ nanoparticles. The maximum decrease in viable counts of *Escherichia coli* bacteria was only 97% while the antibacterial effect was non-monotonous. The Gram-positive bacterium *Staphylococcus aureus* was not affected by nano-CeO_2_ at any of the tested concentrations. Importantly, no cytotoxic effect of nano-CeO_2_ was observed. On the other hand, Ag nanoparticles that have been considered antiviral in several previous reports, did not show any significant antiviral activity in our study, except for a non-enveloped bacteriophage ф6. However, nano-Ag proved antibacterial and reduced the viable counts of bacteria by more than 99.99% already at low mg/l concentrations. At higher concentrations, nano-Ag exhibited also cytotoxic effects. Thus, based on our results we can conclude that Ag nanoparticles exhibit a relatively unspecific biological effect while nano-CeO_2_ show a relatively specific antiviral activity. However, a more detailed research is needed to elucidate the mechanism of antiviral action of CeO_2_ nanoparticles and specific targets of nanoceria in different viruses.

## Materials and methods

All chemicals used in the study were of analytical grade. Components of growth media are described in more detail below.

### Synthesis of CeO_2_, Ag and SiO_2_ nanoparticles

The target materials which antiviral potential was assessed in this study were nanoparticles of ceria that were synthesized using two methods resulting in nano-CeO_2_ with positive and negative surface zeta potential. The antiviral effects of nano-CeO_2_ were compared with nanoparticles of silica (nano-SiO_2_) that were considered biologically inert (negative control) and silver nanoparticles (nano-Ag) that were considered potentially antiviral (positive control). The following chemicals were used for the synthesis of nanoparticles: cerium (III) nitrate hexahydrate (99.0%, Sigma-Aldrich), diammonium cerium (IV) nitrate (98%, Fluka), hexamethylenetetramine (HMTA, analytical grade, Sigma-Aldrich), citric acid (99%, Fluka), sodium citrate dihydrate (98%, Sigma-Aldrich), tetraethyl orthosilicate (TEOS, 98%, Fluka), 0.1 M water solution of AgNO_3_ (Fluka, analytical grade), sodium tetrahydridoborate (Sigma Aldrich, reagent grade), ethyl alcohol (analytical grade, Sigma-Aldrich), polyethylene glycol (PEG, M_w_ 1500, Reagent grade, Sigma Aldrich), polyvinylpyrrolidone (PVP, M_w_ 10000, Reagent grade, Sigma Aldrich), 30% aqueous ammonia solution (Reagent grade, Sigma Aldrich). All solutions were made with MQ deionized water.

For the synthesis of nano-CeO_2_(+) nanoparticles the modified technique from Ref.^74^ was used. Diammonium cerium (IV) nitrate was hydrolyzed at high temperature in the presence of HMTA. For that, 0.189 g of (NH_4_)_2_Ce(NO_3_)_6_ and 0.053 g of HMTA were dissolved in 50 ml of water and loaded into an autoclave vessel (100 ml). The vessel was sealed and heated to 180 °C for 30 min in the microwave-hydrothermal device (Berghof Speedwave 4, 2.45 GHz, 1000 W). After thermal treatment, the vessel was cooled down to room temperature in a water bath. The product was centrifuged for 10 min, the sediment was washed with deionized water and redispersed by ultrasonication. These steps were repeated at least 3 times and the final product was redispersed in 5 ml of MQ water by ultrasonication until opalescent pale-yellow colloid was obtained. As a result, nanoparticles with an almost “bare” surface carrying positive charge were obtained.

For the synthesis of nano-CeO_2_(−) nanoparticles we used a method from Ref.^75^. Cerium(III) nitrate was hydrolyzed at room temperature in the presence of ammonia with simultaneous oxidation by oxygen from the air. For that, 0.045 g of citric acid was dissolved in 25 ml of prepared in advance 0.05 M aqueous solution of cerium (III) nitrate. The solution was rapidly added into 100 ml of 3 M solution of ammonia and left to stir vigorously for 2 h during which the color of solution changed from colorless to yellow-orange. After that the colloid solution was centrifuged, washed and redispersed by ultrasonication. These steps were repeated at least 3 times and the final product was redispersed in 15 ml of MQ water by ultrasonication until transparent dark yellow colloid was obtained. This method allowed the production of ceria nanoparticles stabilized by citrate-ions and therefore carrying a large negative charge.

For synthesis of nano-Ag a two-step seed-mediated technique was used adopted from Ref.^76^ and modified to avoid toxic reagents. For preparation of seeds 20 ml of 2.5 mM solution of sodium citrate were mixed with 1 ml of 5 g/l aqueous PVP (MW 10000) solution and 1.2 ml of freshly prepared 10 mM solution of NaBH_4_. To this mixture 20 ml of 0.5 mM solution of silver nitrate was added at the rate of 2 ml/min under constant stirring. The Ag seeds were left to form for 15 min under stirring at room temperature. In the second stage 27 ml of prepared seed solution was added to the solution of 0.5884 g of sodium citrate in 470 ml of MQ water. This mixture was heated up to 85 °C under stirring and when this temperature was reached, 0.625 ml of 0.1 M AgNO_3_ solution was added to the solution, which was afterwards left under stirring for 15 min. In order to purify and concentrate the obtained solution, ultracentrifugation was performed (Beckman Coulter, Optima XE-90 Ultracentrifuge device, 28,000 rpm/141,000×*g*, 2.5 h). This allowed us to obtain a solution with 0.13 g/ml concentration. From this stock solution, less concentrated samples were prepared by dilution with MQ water.

Synthesis of nano-SiO_2_ was carried out using a modified Stöber technique^77^. For that, 18 ml of MQ water was mixed with 100 ml of ethanol. In this water-alcohol mixture 1.5 g of PEG, 6.2 ml of TEOS and 3 ml of NH_4_OH solution were dissolved. The resulting reaction mixture was left at room temperature under vigorous stirring for 24 h. After that the synthesized SiO_2_ was centrifuged, washed with deionized water and redispersed by ultrasonication. These steps were repeated at least 3 times and the final product redispersed in 5 ml of MQ water by ultrasonication until opalescent colorless colloid was obtained.

### Characterization of CeO_2_, SiO_2_ and Ag nanoparticles

The characteristics of nanoparticles, the size, shape, agglomeration state, size distribution, surface characteristics and solubility, were chosen according to the recommendation by the Scientific Committee on Emerging and Newly Identified Health Risks of the European Commission^79^. Particle size and morphology were studied by means of transmission electronic microscopy (FEI Tecnai F20 TEM). For TEM analysis a drop of, 100 mg/l particle suspension was deposited onto a 400 mesh holey carbon coated copper grid (Agar scientific AGS147-4) and the sample was dried. Particle aggregation potential (hydrodynamic size measurement using dynamic light scattering, DLS) and surface zeta potential measurements were performed using Malvern Zetasizer Nano ZSP from aqueous suspensions of particles at the highest concentration tested in the antiviral assay. In case of nano-CeO_2_, all concentrations tested in antiviral assay were measured using DLS. The measurements of nano-SiO_2_ and nano-Ag were carried out using corresponding pre-set parameters of the device; nano-CeO_2_ measurements were carried out using pre-set parameters for TiO_2_ nanoparticles as the closest among available. Water was used as a solvent in these measurements to resemble the actual exposure condition in antiviral and bacterial assays.

More detailed surface characteristics of nano-CeO_2_ were measured using UV–Vis mode (250–800 nm) of Agilent Cary 5000 UV–Vis–NIR device and FTIR spectral analysis mode of Bruker Vertex 70 FTIR spectrometer. For the latter, ATR configuration with diamond crystal was used, and mercury cadmium telluride and MCT detectors were used. Data was obtained from the range of 4000 cm^−1^ to 400 cm^−1^. Separation of mother solution for elemental analysis was performed using ultracentrifugation (Beckman Coulter, Optima XE-90 Ultracentrifuge device) of colloids at 300,000×*g* for 2 h. Elemental analysis was performed using ICP-MS spectrometer (Agilent Technologies, 2009).

### Toxicity testing of nanoparticles on viral host organisms

Prior to antiviral activity testing, the effects of nanoparticles and other test chemicals were assessed on host organisms of those viruses—cell lines in case of mammalian viruses and bacteria in case of bacteriophages.

#### Cytotoxicity testing with mammalian cell lines

Cytotoxicity was tested against virus host cell lines were tested (Table 4). Cells were seeded onto 96-well plates and grown in specified medium (Table 1) to reach 70% confluency after which growth medium was removed and 100 μl of the tested compound corresponding to that later tested for antiviral effects was added. The cells were incubated for 1 h at 37 °C, 5% CO_2_, humidified atmosphere after which the medium with the test compounds was removed, fresh growth medium was added and cells were further incubated for 48 h. Cell viability was measured using Cell Proliferation Reagent (WST-1) (Roche) tox which 10 μl/well WST-1 reagent was added, the plate was incubated at 37 °C, 5% CO_2_ for 3 h and absorbance at 450 nm was read. For calculation, the value of a blank well (no cells present) was subtracted from each measurement and the value of wells without a test compound was considered as 100% viability. Three replicates were tested for each compound and concentration. The concentrations of test substances that resulted in cellular toxicity were not tested for their antiviral effects as interferences from cellular toxicity could not be avoided.

**Table 4:** Viruses, their host organisms and maintenance conditions.

Virus	Titer in exposure, PFU/ml	Host organism	Maintenance/growth conditions
**Enveloped viruses**			
Mammalian viruses			
SARS-CoV-2	5 × 10^5^	Vero E6 cells (Growth medium: DMEM, 10% heat-inactivated FBS, 100 U/ml penicillin and 100 μg/ml streptomycin; grown at 37 °C and 5% CO_2_)	Virus growth medium: DMEM, 0.2% BSA, 100 U/ml penicillin, 100 μg/ml streptomycin
TGEV	8.35 × 10^6^	ST cells (Growth medium: DMEM, 10% heat-inactivated FBS, 100 U/ml penicillin and 100 μg/ml streptomycin, 37 °C 5% CO_2_)	Virus growth medium: DMEM, 0.2% BSA, 100 U/ml penicillin and 100 μg/ml streptomycin
Influenza virus A/WSN/1933	6.65 × 10^4^	MDCK-2 cells (Growth medium: DMEM, 10% heat-inactivated fetal bovine serum (FBS), 100 U/ml penicillin and 100 μg/ml streptomycin, 37 °C 5% CO_2_)	Virus growth medium: DMEM, 0.2% BSA, 100 U/ml penicillin and 100 μg/ml streptomycin, 1 µg/ml TPCK-treated trypsin
Bacteriophages			
ɸ6	1 × 10^7^	*Pseudomonas* sp. (Growth medium Tryptone Soy Broth TSB: 17 g/l casein peptone, 3 g/l soy peptone, 2.5 g/l glycose, 5 g/l NaCl, 2.5 g/l K_2_HPO_4_; 25 °C; for solid medium 15 g/l agar was added)	Phage maintenance medium: SM buffer (0.1 M NaCl, 8 mM MgSO4, 50 mM Tris–HCl; pH 7.5 Semisolid TSB top agar used for infection: 7 g/l agar added to TSB
**Non-enveloped viruses**			
Mammalian viruses			
EMCV	2.5 × 10^7^	BHK-21 cells (Growth medium: GMEM, 10% heat-inactivated FBS, 2% TPB, 2% 1 M Hepes (pH 7.2), 100 U/ml penicillin, 100 μg/ml streptomycin, 37 °C 5% CO_2_)	Virus growth medium: GMEM, 0.2% BSA, 2% 1 M Hepes (pH 7.2), 100 U/ml penicillin, 100 μg/ml streptomycin
Bacteriophages			
MS2	1 × 10^7^	*Escherichia coli* (Growth medium NZCYM broth: 10 g/l casein hydrolysate, 5 g/l NaCl, 1 g/l casamino acids, 5 g/l yeast extract, 2 g/l MgSO_4_·7H_2_O, 2 g/l maltose 37 °C; for solid medium 15 g/l agar was added)	Phage maintenance medium: SM buffer (see above) Semisolid NZCYM top agar used for infection: 7 g/l agar added to NZCYM

#### Toxicity to host bacteria

Host bacteria for the two bacteriophages (Table 4) were exposed to the test substances in plaque assay format omitting the bacteriophage. Bacterial lawns were created by mixing overnight culture of *Pseudomonas* or exponentially grown culture of *Escherichia coli* with TSB or NZCYM soft agar (Table 4), respectively and poured into Petri dishes as described below under "Bactericidal assays" . 10 μl drops of the specified concentration of test substances (see the tested concentration ranges in Table 2) were pipetted onto the agarized surface. After overnight incubation, bacterial lawn was observed for inhibition zones at the drops of chemicals. None of the nanoparticles or chemicals except nano-Ag resulted in inhibition zones. To mitigate nano-Ag toxicity in antiviral assay with bacteriophages these nanoparticles were tested in combination with threefold molar excess of l-cysteine. Thus, in case of 14 μM nano-Ag equal volume of 42 μM l-cysteine was added, in case of 140 μM nano-Ag equal volume of 420 μM l-cysteine was added and in case of 1400 μM equal volume of nano-Ag 4200 μM l-cysteine was added. l-cysteine concentrations till 4200 μM did not decrease the titer of neither of the bacteriophage.

### Testing of antiviral activity

The six viruses and bacteriophages used in the tests and media used for their infection and propagation are described in Table 4 and below. Nanoparticles and chemicals used for antiviral testing are shown in Table 2. In general, aqueous suspensions of chemicals at specified concentrations were mixed with an equal amount of bacteriophages in water or with mammalian viruses in 1/10 water-diluted cell culture medium. Samples were incubated for 1 h at room temperature after which these were diluted in tenfold series using SM buffer (bacteriophages) or virus growth medium (mammalian viruses). 1 h incubation was chosen to follow the requirements of European Biocidal Products Regulation requirements for antimicrobial efficacy testing of treated articles.

#### Mammalian viruses

Viruses (Tables 2, 4) were obtained from the following sources: SARS-CoV-2 was a local Estonian isolate obtained from Estonian Health Board; influenza virus A/WSN/1933 was from SinoBiological; TGEV was obtained from L. Enjuanes at Department of Molecular and Cell Biology, National Center of Biotechnology (CNB-CSIC), Madrid, Spain; EMCV was obtained from A. Kohl MRC-University of Glasgow Centre for Virus Research, Glasgow, Scotland, United Kingdom. The cell lines for virus propagation (Table 4) were from the cell culture library of Tartu University Institute of Technology, except for ST cells obtained from L. Enjuanes, Madrid, Spain. For propagation of coronaviruses, confluent Vero E6 (for SARS-CoV-2) or ST (for TGEV) cells grown on T75 flask were infected with viral stocks in VGM at a multiplicity of infection 0.01 pfu/cell. Infected cells were incubated for 4 days at 37 °C, 5% CO_2_. Cell supernatant was collected, clarified by centrifugation at 3000×*g* for 15 min at + 4 °C, aliquoted and stored at − 80 °C. EMCV stock was obtained ready to use. For propagation of influenza virus A/WSN/1933, confluent MDCK-2 cells grown on T175 flask were infected in VGM containing TPCK-trypsin at final concentration 1 μg/ml. Infected cells were incubated for 2 days at 37 °C, 5% CO_2_. Cell supernatant was collected, clarified by centrifugation at 4000×*g* for 10 min at + 4 °C, filtered through a 0.2 μm filter, aliquoted and stored at − 80 °C.

For antiviral activity testing, the viruses with initial titers in virus growth media (Table 4) were diluted 1:10 with sterile water, mixed with the test compound or chemical and incubated as above. After incubation, the virus mixtures were diluted with virus growth medium by 10 times and 150 μl of the resulting sample was used to infect 100% confluent host cells grown on 12- or 96-well plates and washed with phosphate buffered saline (PBS). In SARS-CoV-2 experiments, 25 μl of the diluted virus sample was used to infect Vero E6 cells. 150 μl or 25 μl of virus growth medium was added to cells in negative control. Infection was carried out at 37 °C, 5% CO_2_, humidified atmosphere for 1 h with gentle rocking every 10 min. After 1 h, the infection medium was removed and 3:2 mix of virus growth medium: 2% carboxymethyl cellulose (CMC) was added (in case of influenza virus the mix was supplemented with 1 µg/ml TPCK-trypsin). Cells were grown in respective growth media for 96 h (as an exception 48 h in case of EMCV and Vero E6) at 37 °C, 5% CO_2_, humidified atmosphere. For plaque counting in ST, MDCK-2 and BHK-21 cells, the virus growth medium was removed, and plates were dyed using crystal violet. Plaques were identified as clear plaques within the cell layer. In Vero E6 cells, mini-plaque method was used for which plates were fixed with ice-cold 80% acetone in 1× PBS at − 20 °C for 1 h. Acetone was then removed by pipetting and the plates were dried for at least 3 h. Dried plates were treated with a blocking buffer (Thermo Scientific, Ref. 37587) diluted in PBS/0.05% Tween20 (PBS-T) for 60 min at 37 °C. Next, the plates were probed with rabbit monoclonal anti-SARS-CoV-2 nucleocapsid antibody 82C3 (Icosagen AS, ref. nr. R1-166-100) diluted in PBS-T for 1 h at 37 °C. The plates were washed 6 × 5 min with PBS-T and treated with secondary goat anti-rabbit IRDye800CW antibody (LI-COR Biosciences, ref. nr. 926-32211) diluted in blocking buffer for 60 min at 37 °C. Plates were washed with PBS-T 6 × 5 min, dried and scanned using LI-COR Biosciences Odyssey Infrared Imaging System and application software, to identify fluorescent focuses (mini-plaques). Minimum 3 plaques and maximum of 30 plaques per well were counted and PFU value was calculated according to the dilution and the volume of the inoculum. Each concentration of nanoparticles or salt was tested in three replicates and at least three independent experiments were performed.

Antiviral activity was calculated as a difference between log-transformed plaque forming unit (PFU) counts in negative control and the test sample. 2-log decrease in PFU counts during 1 h was considered as the lowest biologically meaningful threshold in potential applications.

#### Bacteriophages

The enveloped bacteriophage ф6 (DSM21518) and non-enveloped MS2 (DSM21428) and their respective host bacteria, *Pseudomonas* sp. (DSM21428) and *Escherichia coli* (DSM5695) were from German Collection of Microorganisms and Cell Cultures (DSMZ). Bacteriophages were propagated in their host bacteria and purified in titers shown in Table 4. Bacteriophages in water were exposed to compounds and chemicals as above for 1 h after which SM buffer was used to dilute phages for plaque assay. For infection, 10 μl of bacteriophage samples were dropped as ~ 0.5 cm wide circles onto bacterial plates prepared from overnight grown culture (*Pseudomonas* sp.) or 2 h grown exponential culture prepared from overnight culture (*Escherichia coli*). 125 μl of *E. coli *or *Pseudomona*s *sp.* were mixed with 5 ml of semisolid top agar medium (0.7% agar in NZCYM or TSB, respectively at 40 °C and poured onto solid lysogeny broth (LB: 10 g/l tryptone, 5 g/l yeast extract, 10 g/l NaCl) agar plates (1.5% agar) as a thin layer. 10 μl of phage that was incubated in water without compounds as dropped onto bacterial lawns for negative control. Plaques (areas with no visible growth of the host bacterium) within the circles were counted after overnight incubation to obtain the plaque forming units PFU/ml. Minimum of 3 plaques were counted per circle. Each concentration of nanoparticles or chemicals was tested with two technical replicates and at least three independent experiments were performed.

### Bactericidal assays

In antibacterial tests *Escherichia coli* DSM1576 (ATCC8739) and *Staphylococcus aureus (*ATCC6538) originating from American Type Culture Collection were used. Nanoparticles and Ce(NO_3_)_3_ at specified concentrations were mixed with an equal volume of bacterial suspensions in water. Bacterial suspensions with 1 × 10^7^ CFU/ml were prepared by cultivating the bacteria in their growth media (LB) and subsequent washing with water (5 min centrifugation, resuspension in water and following centrifugation). Bacteria with specified concentrations of nanoparticles and chemicals were incubated for 1 h at room temperature after which the samples were diluted in tenfold series using PBS and drop-plated for viable cell counting (colonies were counted after overnight growth) as in Ref.^78^. Minimum of 3 colonies were counted per drop. Each condition was tested with two technical replicates and at least three experiments were performed. Antibacterial activity was calculated as a difference between log-transformed colony forming units (CFU) in negative control and the test sample.

### ELISA assay to assess receptor binding of SARS-CoV-2 in the presence of nano-CeO_2_

Binding of nano-CeO_2_ particles to virus surface proteins was analyzed using SARS-CoV-2 and its cellular receptor human recombinant ACE2-hFc. Maxisorp (Nunc) ELISA plates were covered with ACE2-hFc (product P-308-100, Icosagen, Estonia) in PBS at 1 μg/well, and incubated at + 4 °C overnight. The plate was washed with PBS-T (phosphate-buffered saline with addition of 0.05% Tween20) 5 × 5 min, blocked with 3% BSA/PBS at + 4 °C overnight and then washed with PBS-T. 10 μl of nano-CeO_2_(+) and (−) at varying concentrations was mixed with 10 μl (5 × 10^4^ PFU, i.e., 5 × 10^5^ PFU/ml) of recombinant SARS-CoV-2 (rescued from infectious clone constructed at the Tartu University Institute of Technology; contains mutations in SARS-CoV-2 S-protein from South African isolate). Mixtures were incubated at room temperature for 1 h. 4 μl of virus-nanoparticle mixture was diluted 25 times and transferred to the plate coated with recombinant hACE2 receptor. The plate was incubated at 37 °C overnight, fixed with ice-cold 80% acetone/PBS at − 20 °C for 1 h. Acetone was removed and the plate dried for 1 h. After that blocking with 3% BSA/PBS was carried out at + 37 °C for 1 h. Primary antibody, rabbit monoclonal antibody against SARS-CoV2 RBD 72A5 B12 (R1-172-100 Icosagen, Estonia) was diluted in 3% BSA/PBS-T 1:5000 and placed onto the ELISA plate at + 4 °C overnight. Then, the plate was washed with PBS-T 5 × 5 min and incubated with secondary antibody, anti-rabbit-HRP (horseradish peroxidase conjugate, LabAS, Tartu, Estonia) diluted 1:10,000 in 3% BSA/PBS-T at + 37 °C for 1 h. The plate was then washed with PBS-T 5 × 5 min and 50 μl/well of 3,3ʹ,5,5ʹ-Tetramethylbenzidine substrate (Bio-Rad) was added, the wells were incubated at room temperature for 15 min. The reaction was stopped by adding 50 μl/well of 0.5 M H_2_SO_4._ Absorbance at 450 nm was quantified using a microplate reader. Wells non-treated with hACE2 were used as negative control.

### Statistical analysis

Log-transformed data was used for the analysis of PFU or CFU counts. Two-way ANOVA analysis followed by Dunnett’s post-hoc test to detect significant differences from control and corrected for multiple comparisons at 0.05 significance level was executed in GraphPad Prism 9.3.0. Only statistically significant differences are marked in Table 3.

## Supplementary Information

Supplementary Figures.

## Data Availability

The original data are available upon request from the corresponding author: angela.ivask@ut.ee.
