# Supplementary material for: Antiviral efficacy of cerium oxide nanoparticles
Source: Sci Rep. 2022 Nov 5;12:18746. doi: 10.1038/s41598-022-23465-6 (PMC9637224; doi:10.1038/s41598-022-23465-6)
Supplement: Supplementary file 1 — Supplementary Figures. [file 41598_2022_23465_MOESM1_ESM.pdf]

Supplementary material for

Antiviral efficacy of cerium oxide nanoparticles

Alexandra Nefedova[1*], Kai Rausalu[2*], Eva Zusinaite[2], Alexander Vanetsev[1], Merilin Rosenberg[3,4], Kairi Koppel[3], Stevin Lilla[1], Meeri Visnapuu[1], Krisjanis Smits [5], Vambola Kisand[1], Tanel Tätte[1], Angela Ivask[3**]

[1] Institute of Physics, University of Tartu, W. Ostwaldi Street 1, 50411, Tartu, Estonia

[2] Institute of Technology, University of Tartu, Nooruse Street 1, 50411, Tartu, Estonia

[3] Institute of Molecular and Cell Biology, University of Tartu, Riia Street 23, 51010, Tartu, Estonia

[4] Department of Chemistry and Biotechnology, Tallinn University of Technology, Akadeemia Street 15, 12618, Tallinn, Estonia

[5] Institute of Solid State Physics, University of Latvia, Kengaraga Street 8, LV-1063, Riga, Latvia

*Shared first authorship

** Corresponding author: angela.ivask@ut.ee

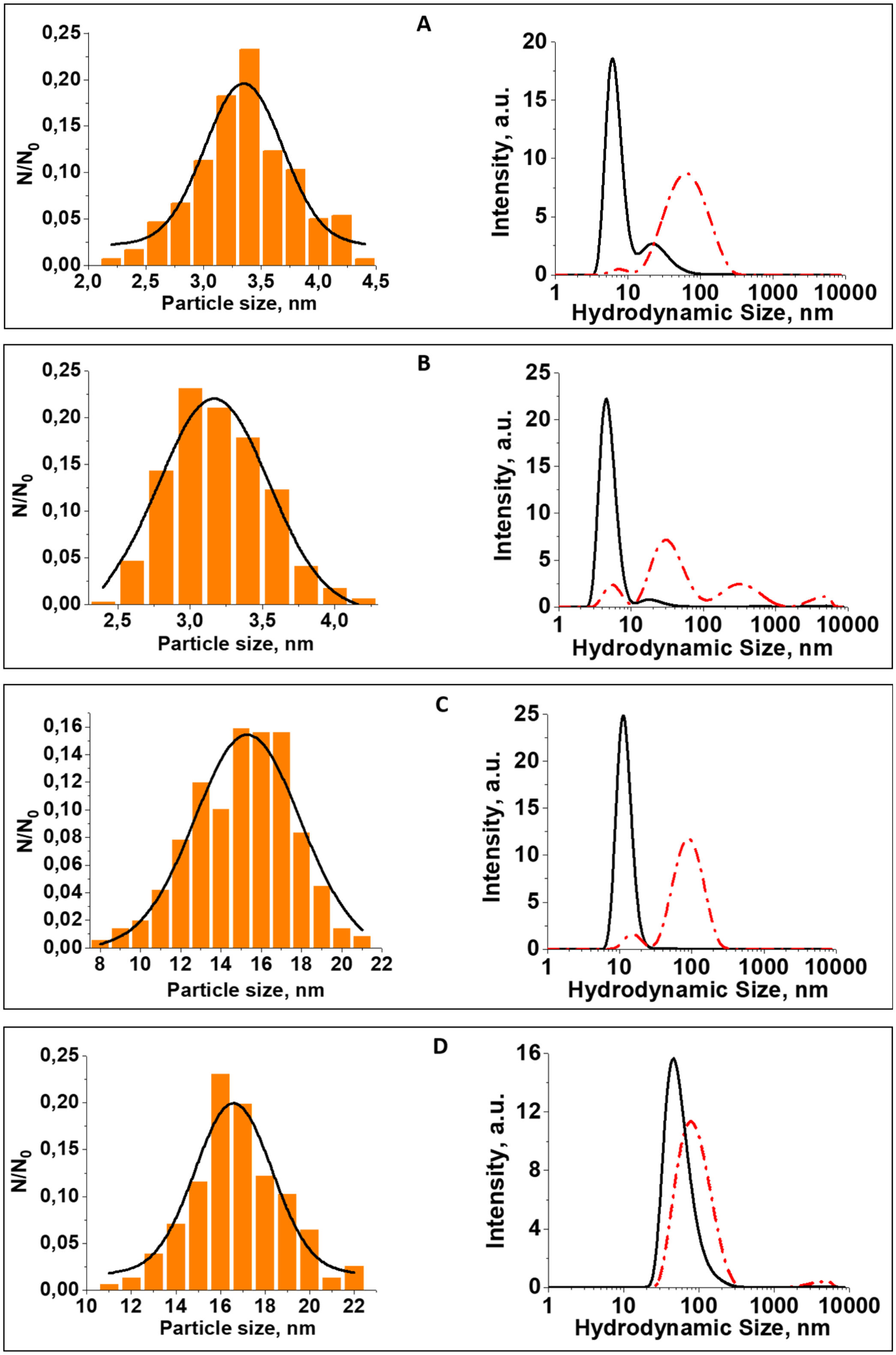

Figure S1. Particle size distribution as calculated from HRTEM images (left column) and from DLS data (right column).

A: nano-CeO_2(+), B: nano-CeO_2(-), C: nano-Ag, D: nano-SiO_2. On DLS graphs the solid line is a distribution by the

number of particles and dashed line by intensity of scattering. HRTEM data is based on measuring the diameter of >300 particles except for SiO_2 (under 200 was measured due to complications with image analysis).

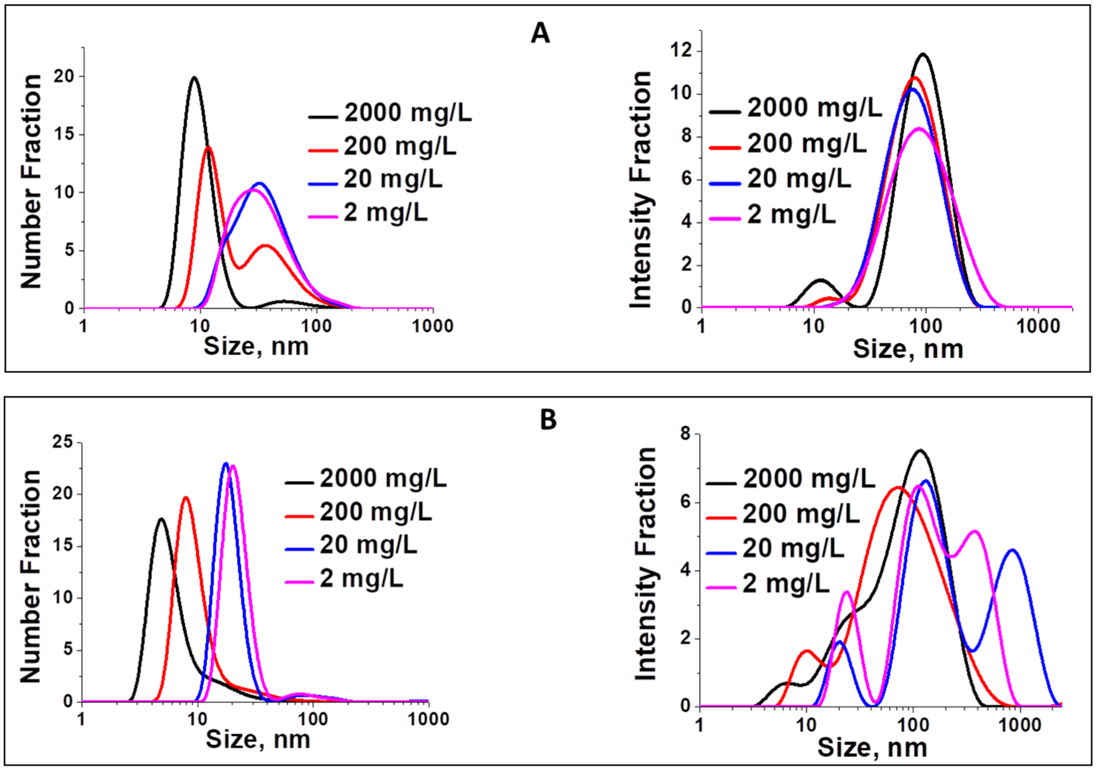

Figure S2. Evolution of hydrodynamic particle size with changing concentration of nano-CeO_2(+) and (-) according to number fraction (left side) and intensity fraction (right side). The dilution of both nano-CeO_2(+) and nano-CeO_2(-) colloids leads to aggregation of individual particles and disappearance of single nanoparticles. The aggregation is monotonous in case of nano-CeO_2(+) but in case of nano-CeO_2(-) the particles behavior is more complex. While individual particles also tend to aggregate monotonously with the dilution, the size distribution of the final aggregates in these colloids is broad and multiband and changes in a non-linear manner.

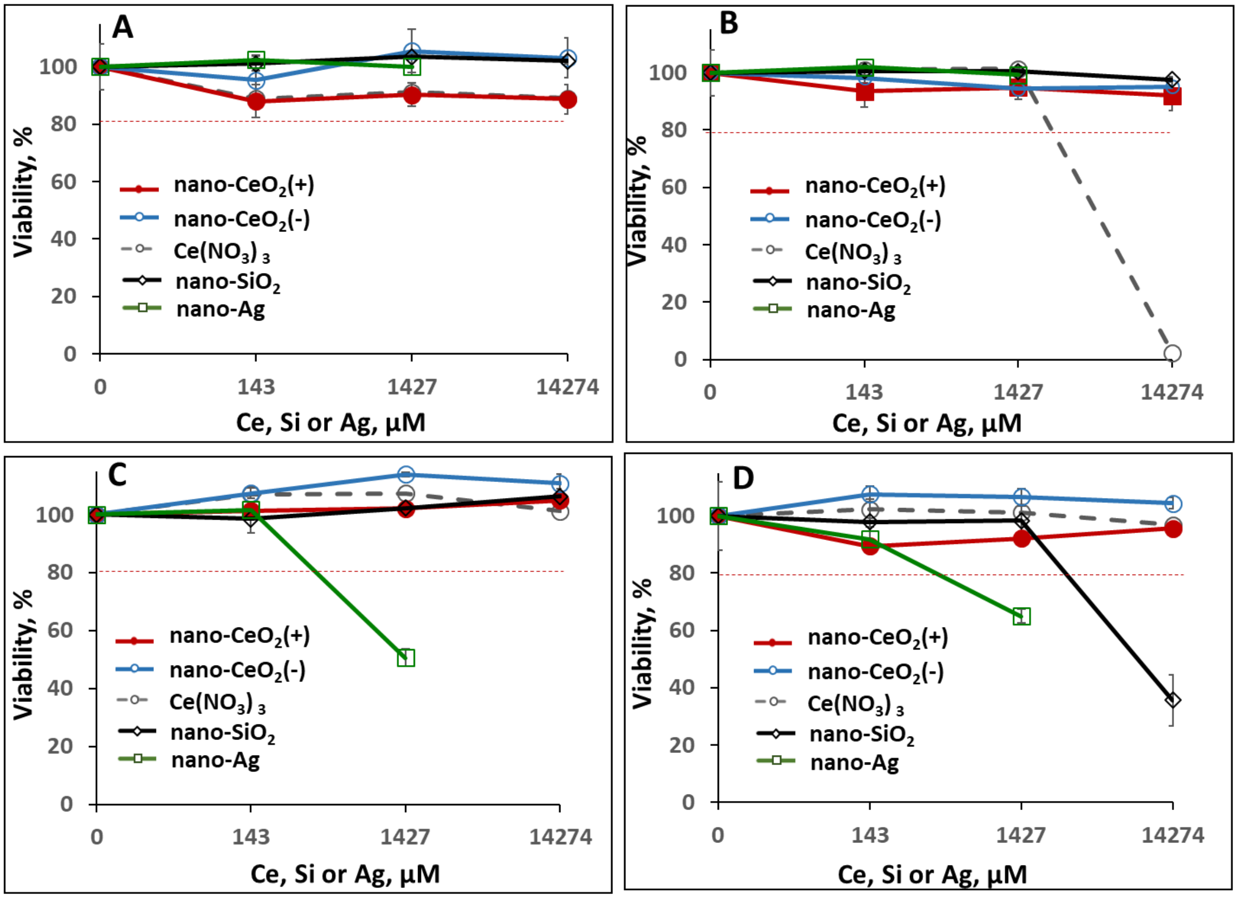

Figure S3. The effect of tested nanoparticles and chemicals on viability of host cells of mammalian viruses. A: BHK-21 cells used as hosts for EMCV virus, B: Vero-E6 cells used as host for SARS-CoV-2 virus, C: MDCK-2 cells used as a host for A/WSN/1933 influenza virus, D: ST cells used as a host for TGEV virus. Average cell viability results (% viability compared with non-exposed cells) of three experiments with standard deviation are shown. Decrease of cellular viability to less than 80% (red dotted line) was considered cytotoxic. To enable comparison between compounds and with Figure 6, the concentrations of tested compounds are shown in µM. nano-Ag could only be analyzed until 1427 µM as the highest concentration of the synthesized particles was 4400 µM.

During antiviral testing, the concentration of nanoparticles and $Ce(NO_3)_3$ that was exposed to viral host cells in infection procedure was 10-fold lower compared with the concentrations of those compounds and nanoparticles that was exposed to viruses.

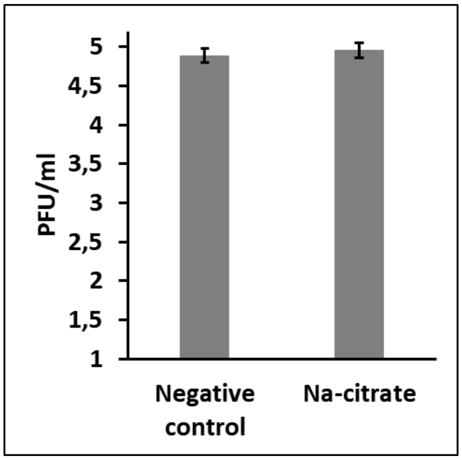

Figure S4. The effect of citric ions on PFU/ml of A/WSN/1933 influenza virus. The tested sodium citrate concentration was 7.6 mM that equals with the concentration of sodium citrate used in silver nanoparticles synthesis and approximately 5 times higher than citric acid concentration used during nano-CeO_2(-) particles synthesis process. The average of two separate experiments is shown.
